# G protein-coupled estrogen receptor inhibits the P2Y receptor-mediated Ca^2+^ signaling pathway in human airway epithelia

**DOI:** 10.1007/s00424-016-1840-7

**Published:** 2016-06-06

**Authors:** Yuan Hao, Alison W. Chow, Wallace C. Yip, Chi H. Li, Tai F. Wan, Benjamin C. Tong, King H. Cheung, Wood Y. Chan, Yangchao Chen, Christopher H. Cheng, Wing H. Ko

**Affiliations:** School of Biomedical Sciences, The Chinese University of Hong Kong, Shatin, Hong Kong; School of Biomedical Sciences, The University of Hong Kong, Pokfulam, Hong Kong

**Keywords:** GPER, P2Y receptor signaling pathway, Human bronchial epithelial cells, Calcium signaling, cAMP

## Abstract

P2Y receptor activation causes the release of inflammatory cytokines in the bronchial epithelium, whereas G protein-coupled estrogen receptor (GPER), a novel estrogen (E_2_) receptor, may play an anti-inflammatory role in this process. We investigated the cellular mechanisms underlying the inhibitory effect of GPER activation on the P2Y receptor-mediated Ca^2+^ signaling pathway and cytokine production in airway epithelia. Expression of GPER in primary human bronchial epithelial (HBE) or 16HBE14o- cells was confirmed on both the mRNA and protein levels. Stimulation of HBE or 16HBE14o- cells with E_2_ or G1, a specific agonist of GPER, attenuated the nucleotide-evoked increases in [Ca^2+^]_i_, whereas this effect was reversed by G15, a GPER-specific antagonist. G1 inhibited the secretion of two proinflammatory cytokines, interleukin (IL)-6 and IL-8, in cells stimulated by adenosine 5′-(γ-thio)triphosphate (ATPγS). G1 stimulated a real-time increase in cAMP levels in 16HBE14o- cells, which could be inhibited by adenylyl cyclase inhibitors. The inhibitory effects of E_2_ or G1 on P2Y receptor-induced increases in Ca^2+^ were reversed by treating the cells with a protein kinase A (PKA) inhibitor. These results demonstrated that the inhibitory effects of G1 or E_2_ on P2Y receptor-mediated Ca^2+^ mobilization and cytokine secretion were due to GPER-mediated activation of a cAMP-dependent PKA pathway. This study has reported, for the first time, the expression and function of GPER as an anti-inflammatory component in human bronchial epithelia, which may mediate through its opposing effects on the pro‐inflammatory pathway activated by the P2Y receptors in inflamed airway epithelia.

**Electronic supplementary material:**

The online version of this article (doi:10.1007/s00424-016-1840-7) contains supplementary material, which is available to authorized users.

## Introduction

Estrogen (E_2_) is an important hormone that protects the lungs from inflammatory damage. Clinical observations suggested that reduced E_2_ levels were associated with greater risks of lung pathologies in menopausal women [19, 36]. However, the detailed anti-inflammatory role played by E_2_ and its pathophysiological mechanism are still unknown. In addition to the classical nuclear hormone receptors, ERα and ERβ, a novel E_2_ receptor and a G protein-coupled estrogen receptor (GPER), were recently identified [35, 43]. Despite the accumulating body of evidence indicating that the rapid, nongenomic actions of E_2_ observed in the epithelia are mediated via GPER, few studies have investigated the specific role of GPER in inflammatory airway diseases [31, 32].

Extracellular nucleotide release and the subsequent activation of P2Y receptors have been implicated in the pathogenesis of several inflammatory lung disorders, such as asthma [28]. During airway inflammation, damage to the surface epithelium is due to the secretion of eosinophil-derived, highly toxic, cationic proteins, such as major basic protein (MBP). Our recent study demonstrated that when human bronchial surface epithelia are chemically damaged by poly-l-arginine as a surrogate of MBP [10], nucleotides, such as ATP and UDP, are released into the extracellular medium. The extracellular nucleotides then activate cell surface P2Y receptors to release two proinflammatory cytokines, interleukin (IL)-6 and IL-8, via a Ca^2+^-dependent process [20].

To the best of our knowledge, no reports have determined whether GPER is expressed in airway epithelia or whether GPER plays a role in the regulation of P2Y receptor-mediated Ca^2+^ signaling and cytokine secretion in airway epithelia. Therefore, we examined the expression of GPER and its subcellular localization in human bronchial epithelia. We also characterized the cross talk between the GPER and P2Y receptor signaling pathways and its implications on the anti-inflammatory role of GPER.

## Materials and methods

### Solutions and chemicals

Krebs-Henseleit (KH) solution and the nominally Ca^2+^-free solution were prepared as previously described [24]. Membrane permeant acetoxymethyl (AM) ester forms of Fura-2 and Pluronic F127 were obtained from Invitrogen (Carlsbad, USA). Uridine 5′-triphosphate (UTP), uridine 5′-diphosphate (UDP), adenosine 5′-(γ-thio)triphosphate (ATPγS), forskolin, poly-l-arginine hydrochloride, SQ 22536, U73122, E_2_, and G1 were obtained from Sigma-Aldrich (St. Louis, USA). G15 was obtained from Tocris (Bristol, UK). H89 dihydrochloride and MDL 12330A were obtained from Calbiochem (La Jolla, USA). All other general laboratory reagents were obtained from Sigma-Aldrich, and all cell culture reagents were obtained from Invitrogen.

### Cell culture

The 16HBE14o- cell line was maintained in minimum essential medium (MEM) supplemented with 10 % fetal bovine serum, 1 % penicillin/streptomycin, and 1 % glutamine (Invitrogen, Carlsbad, CA) and cultured as described previously [47]. In some experiments, MEM with no phenol red (Invitrogen) was applied. Primary HBE cells were obtained from ScienCell Research Laboratories (Carlsbad, USA) and cultured using Bronchial Epithelial Cell Medium (ScienCell Research Laboratories) following the commercial protocol described previously [20].

### RNA extraction, reverse transcription PCR, and real-time PCR

Total RNA was extracted with TRIzol Reagent (Invitrogen) and reverse transcribed to cDNA using iScript™ Reverse Transcription Supermix (Bio-Rad Laboratories, Hercules, USA). Reverse transcription PCR (RT-PCR) was performed with TaKaRa Taq™ DNA polymerase. Real-time PCR was performed with an Applied Biosystems Power SYBR Green PCR Master Mix (Invitrogen) on a ViiA™ 7 real-time PCR system. GPER primer sequences were as follows: forward primer, 5′-TCTACACCATCTTCCTCTTCC-3′; and reverse primer, 5′-GTAGCGGTCGAAGCTCATCC-3′. The RT-PCR products were characterized using 2 % agarose gel electrophoresis. Relative expression of GPER was normalized to GAPDH and determined with the Pfaffl method [30]. Each run of PCR included a nontemplate control and a sample without reverse transcriptase.

### Western blotting

Western blotting was performed as described previously [10]. Cells grown in culture dishes were lysed on ice in Cytobuster™ Protein Extraction Reagent (Merck Millipore, Billerica, USA), supplemented with a protease inhibitor cocktail (no. 78429, Thermofisher Scientific, Waltham, USA) and a phosphatase inhibitor cocktail (Merck Millipore). Protein samples (20 μg per lane) were transferred to polyvinylidene fluoride (PVDF) membranes (Immobilon-P, Merck Millipore) and immunoblotted with GPER rabbit polyclonal antibody (N-15)-R (1:500; Santa Cruz Biotechnology, Santa Cruz, USA). Blocking peptide (sc-38525 P, Santa Cruz Biotechnology) was used for GPER antibody preabsorption, and mouse monoclonal antibody to GAPDH was used as a loading control. All blots were developed using an enhanced chemiluminescence detection system (Merck Millipore). The apparent molecular masses were calculated using prestained sodium dodecyl sulfate polyacrylamide gel electrophoresis (SDS-PAGE) midrange protein markers (no. HM0671, Hou-Bio Life Technologies, Hong Kong).

### Immunofluorescence microscopy

16HBE14o- or primary HBE cells grown on coverslips in four-well plates were rinsed with phosphate-buffered saline (PBS) and fixed in 4 % paraformaldehyde for 10 min at room temperature. Cells were blocked using PBS with 10 % normal horse serum and 0.1 % Triton X-100 for 1 h and incubated with GPER (N-15)-R rabbit polyclonal antibody (1:60; sc-48525-R, Santa Cruz Biotechnology) overnight at 4 °C [13]. After washing, Alexa Fluor® 488 donkey anti-rabbit IgG (H+L) was added (1:300, Thermofisher Scientific, Waltham, USA). The coverslips were mounted using mounting medium with 1.5 μg/ml 4′,6-diamidino-2-phenylindole (DAPI). Images were captured using a FluoView™-FV1000 confocal microscope (Olympus, Center Valley, USA). In some experiments, cells were co-incubated with purified mouse anti-E-cadherin (1:200; no. 610181, BD Biosciences, Heidelberg, Germany), purified mouse anti-GM130 (1:200; no. 610823, BD Biosciences), or KDEL antibody (1:500; NBP1-97469, Novus Biologicals, Littleton, USA). Alexa Fluor® 555 donkey anti-rabbit IgG was used for visualization (1:400, Thermofisher Scientific). For the negative control group, GPER antibodies were preabsorbed with specific blocking peptides (sc-48525 P, Santa Cruz Biotechnology).

### Small interfering RNA lentivirus packaging and transduction

Lentiviral transfer vectors containing small interfering RNA (siRNA)-targeting GPER were purchased from Applied Biological Materials Inc. (Canada). A lentiviral vector with a scramble siRNA sequence was used as the negative control. The VSV-G-pseudotyped lentiviruses were produced by co-transfecting 293T cells with the transfer vectors and three packaging vectors, pMDLg/pRRE, pRSV-REV, and pCMV-VSVG, by calcium phosphate transfection. At 72 h post-transfection, the cell culture supernatant was collected and filtered through a 0.4-μm filter. The lentivirus was concentrated with centrifugation at 20,000 rpm and resuspended in 1× Tris-buffered saline. For lentiviral transduction, 5 × 10^3^ cells were seeded in 24-well plates, and lentivirus was added to the cells in the presence of 8 μg/ml hexadimethrine bromide (Sigma-Aldrich, St. Louis, USA) overnight. After puromycin selection, the knockdown efficiency of GPER expression was determined by real-time PCR and Western blot analysis.

### Measurement of intracellular calcium concentrations

Calcium signals in cells grown on glass coverslips were measured as previously described [47, 48]. Fura-2 ratios were used to represent changes in [Ca^2+^]_i_ using *Felix* software (Photon Technology International, Edison, USA ). In Ca^2+^ imaging experiments, the perfusion chamber was mounted on an inverted microscope (Olympus IX70, USA) equipped with a scientific CMOS camera (pco.edge 5.5; PCO AG, Kelheim, Germany). Images were digitized and analyzed using MetaFluor Imaging Software (v7.5, Molecular Devices, USA). The data were also shown quantitatively as a change in Fura-2 ratios.

### Manganese quenching

The manganese quench technique was used to estimate calcium influx [15, 45]. 16HBE14o- cells were loaded with Fura-2 as previously described. Since Mn^2+^ has a similar permeability as Ca^2+^ through most plasma membrane Ca^2+^ channels and quenches Fura-2 fluorescence at all excitation wavelengths, Ca^2+^ influx can be estimated by the Mn^2+^ quench of Fura-2 fluorescence at the Ca^2+^-insensitive 360-nm excitation wavelength. During the measurement, cells were treated with 10-μM UTP in the absence (nucleotide alone) or presence of E_2_ (100 nM) or G1 (10 nM) for 10 min. Then 1-mM MnCl_2_ was added in perfusion solution to observe the extent of Mn^2+^ entry. The rate of Mn^2+^ quenching was assessed by measuring the change of slope of Fura-2 fluorescence decrease before and after the addition of Mn^2+^ application (using Originlab 8 software, Northampton, USA), as well as the percentage decrease of Fura-2 fluorescence 120 s after Mn^2+^ application [6, 29, 41].

### Monitoring STIM1 oligomerization via FRET microscopy

FRET microscopy was used to monitor the dynamic oligomerization of stromal interaction molecule 1 (STIM1) in 16HBE14o- cells. Plasmids expressing N-terminally tagged cyan fluorescent protein (CFP)- and yellow fluorescent protein (YFP)-STIM1, pEX-SP-CFP-STIM1, and pEX-SP-YFP-STIM1 were obtained from Addgene (Cambridge, USA). Cells were incubated in Ca^2+^-free Hanks’ Balanced Salt Solution (HBSS) with 4-(2-hydroxyethyl)-1-piperazineethanesulfonic acid (HEPES) during imaging experiments. E_2_, G1, or dimethyl sulfoxide (DMSO) vehicle in HEPES-HBSS modified was perfused into the imaging chamber using a perfusion pencil (AutoMate Scientific, Berkeley, USA) from 0 to 15 min, followed by the addition of ATP to the perfusion solution to deplete Ca^2+^ in the ER. Images were captured using a Nikon Eclipse T*i* microscope with a SPOTS RT3 camera (SPOT Imaging Solutions, Sterling Heights, USA). An ND8 filter was used during the experiment to reduce photobleaching. Each set of images (CFP, YFP, and FRET channels) was taken at 0.2 Hz using MetaFluor 7.8 software. The captured images were analyzed with ImageJ software (NIH, Bethesda, USA) using the pixel-to-pixel comparison method [16]. Sensitized emission was employed for the bleed-through correction. These specific bleed-throughs were obtained on a pixel-to-pixel basis from cells transfected with CFP-STIM1 or YFP-STIM1 alone. In our experimental settings, specific bleed-throughs for CFP and YFP were 0.696 and 0.064, respectively. To reduce photobleaching errors, *N*_*FRET*_ measurements were adopted as described [49].

### Quantitative measurement of STIM1 puncta formation

The formation STIM1 puncta after ER Ca^2+^ depletion by ATP stimulation was measured as described [18]. In brief, 16HBE14o- cells were transfected with YFP-STIM1 (Addgene, Cambridge, USA). Transfected cells were stimulated with ATP (10 μM) in the absence of extracellular Ca^2+^ in HEPES-HBSS solution. Single and isolated cells were selected for analyses. Z-stacks of images were acquired in a 0.225-μm separation. A Z-slice image near the close proximity to the attachment surface was selected for puncta analysis (~225 nm thickness) by ImageJ (NIH). YFP puncta were determined by particle analysis plugin, and fluorescent intensity greater than the background by three standard deviations was measured. Those with size less than 0.2 μm^2^ and greater than 2.5 μm^2^ were excluded for the analyses. In some experiments, transfected cells were pretreated with G1 (10 nM) or E_2_ (100 nM) before stimulation with ATP.

### Real-time measurement of cAMP levels

CFP-Epac-YFP, an Epac-based polypeptide FRET reporter [46], was used to monitor real-time cyclic adenosine monophosphate (cAMP) changes in 16HBE14o- cells. The experiments were performed using the MetaFluor Imaging system (with the FRET module). Cells were transfected with the Epac-based cAMP sensor and excited at 436-nm wavelengths. CFP and YFP images were simultaneously recorded by the imaging setup equipped with the photometrics DV^2^ emission splitting system (Photometrics, Tucson, USA) including two emission filters (470/30 nm for CFP; 535/30 nm for FRET). Acquired fluorescence images were background subtracted, and real-time cAMP levels were represented by normalizing the CFP/FRET emission ratios as described previously [20, 24]. Images were digitized and analyzed using MetaFluor imaging software.

### Inositol-1-phosphate measurements

16HBE14o- cells were plated 24 h before the experiment into 24-well culture plates at a concentration of 5 × 10^4^ cells/well. Agonist-induced inositol-1-phosphate (IP_1_) accumulation in 16HBE14o- cells was quantified using the Cisbio IP-One kit (Cisbio Bioassays, Codolet, France) according to the manufacturer’s instructions [51].

### Quantification of IL-6 and IL-8 secretion

Quantification of IL-6 and IL-8 secretion was performed using an enzyme-linked immunosorbent assay (ELISA) [10]. Cells were grown in 24-well culture plates. Cell-free supernatants were collected from control and treated cells and analyzed using a commercially available ELISA kit specific for IL-6 (eBioscience, San Diego, USA) and IL-8 (BD Biosciences, San Diego, USA) according to the manufacturers’ protocols. All experiments were performed in duplicate.

### Statistical analysis

Data were expressed as the mean ± the standard error of the mean (SEM), and values of *n* referred to the number of independent experiments for each group. Statistical comparisons between original data were performed using the Student’s *t* test and analysis of variance (ANOVA) where appropriate. *P* < 0.05 was considered to be statistically significant.

## Results

### Expression and subcellular localization of GPER in human bronchial epithelial cells

**Fig. 1:**
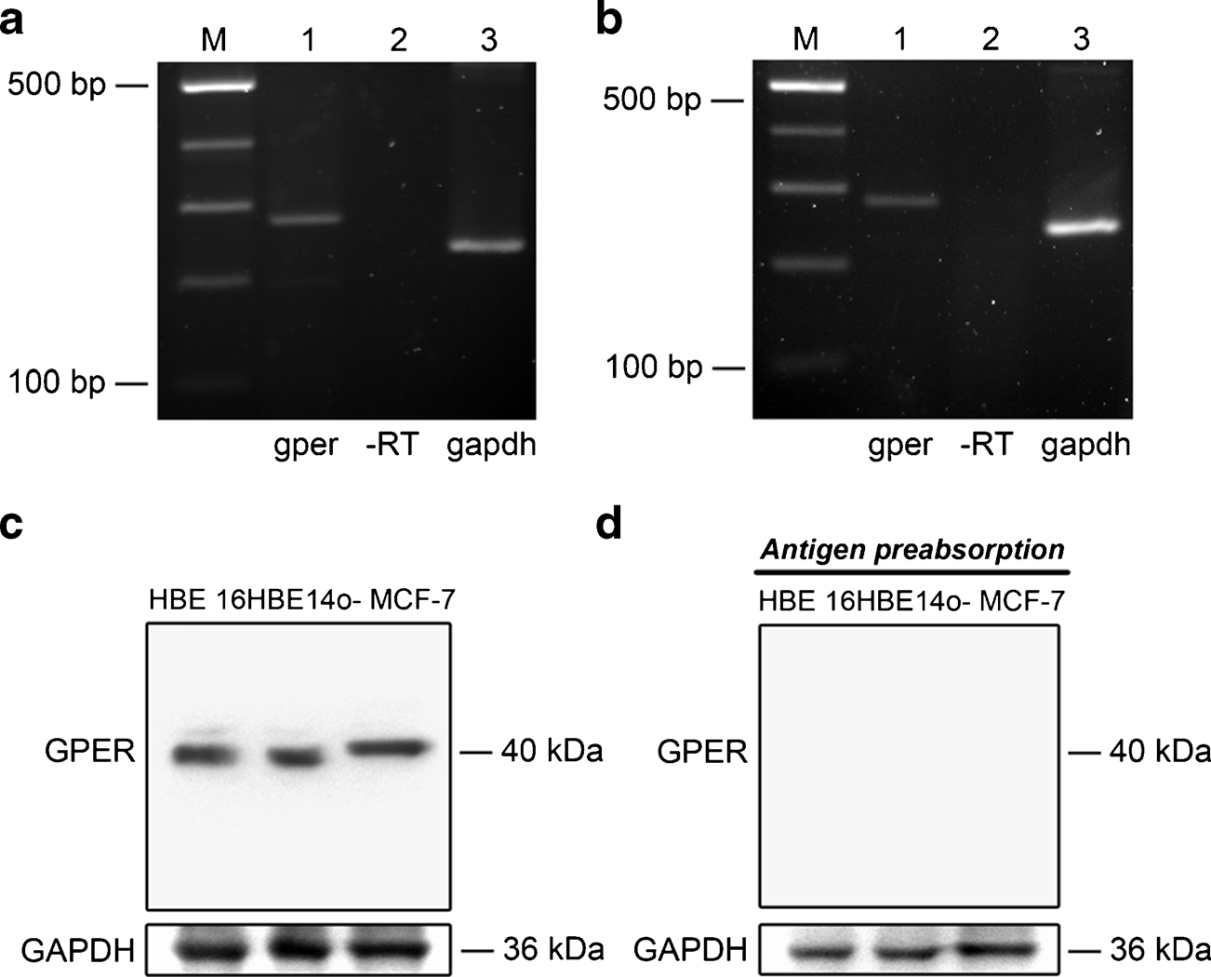
GPER expression in HBE cells. **a**, **b** Agarose gel electrophoresis of the *gper* RT-PCR product obtained from 16HBE14o- (**a**) and primary HBE (**b**) cells, respectively. *Lane 1*, *gper* PCR product (278 bp); *lane 2*, RT negative control product; *lane 3*, *Gapdh* housekeeping PCR product (236 bp); *M*, DNA markers (no. 3422A, Takara Bio Inc., Tokyo, Japan) (*n* = 3–4). **c** GPER protein bands (40 kDa) in primary HBE cells, 16HBE14o- cells, and MCF-7 cells (positive control). **d** GPER antibody was blocked by peptide preabsorption. GAPDH (36 kDa) was used as an internal control (*n* = 3)

RT-PCR was performed to assess *gper* messenger RNA (mRNA) expression in 16HBE14o- (Fig. 1a) and primary human bronchial epithelial (HBE; Fig. 1b) cells. The PCR product of *gper* mRNA was expressed in both cell types (lane 1), and GPER protein expression was examined by Western blotting. GPER was detected as a single protein band of approximately 40 kDa in both primary HBE and 16HBE14o- cells (Fig. 1c). MCF-7 cells were used as a GPER-positive cell line [3]. Detection of these protein bands was specific, as they were abolished by prior preabsorption of the antibodies with a control antigen (Fig. 1d).

**Fig. 2:**
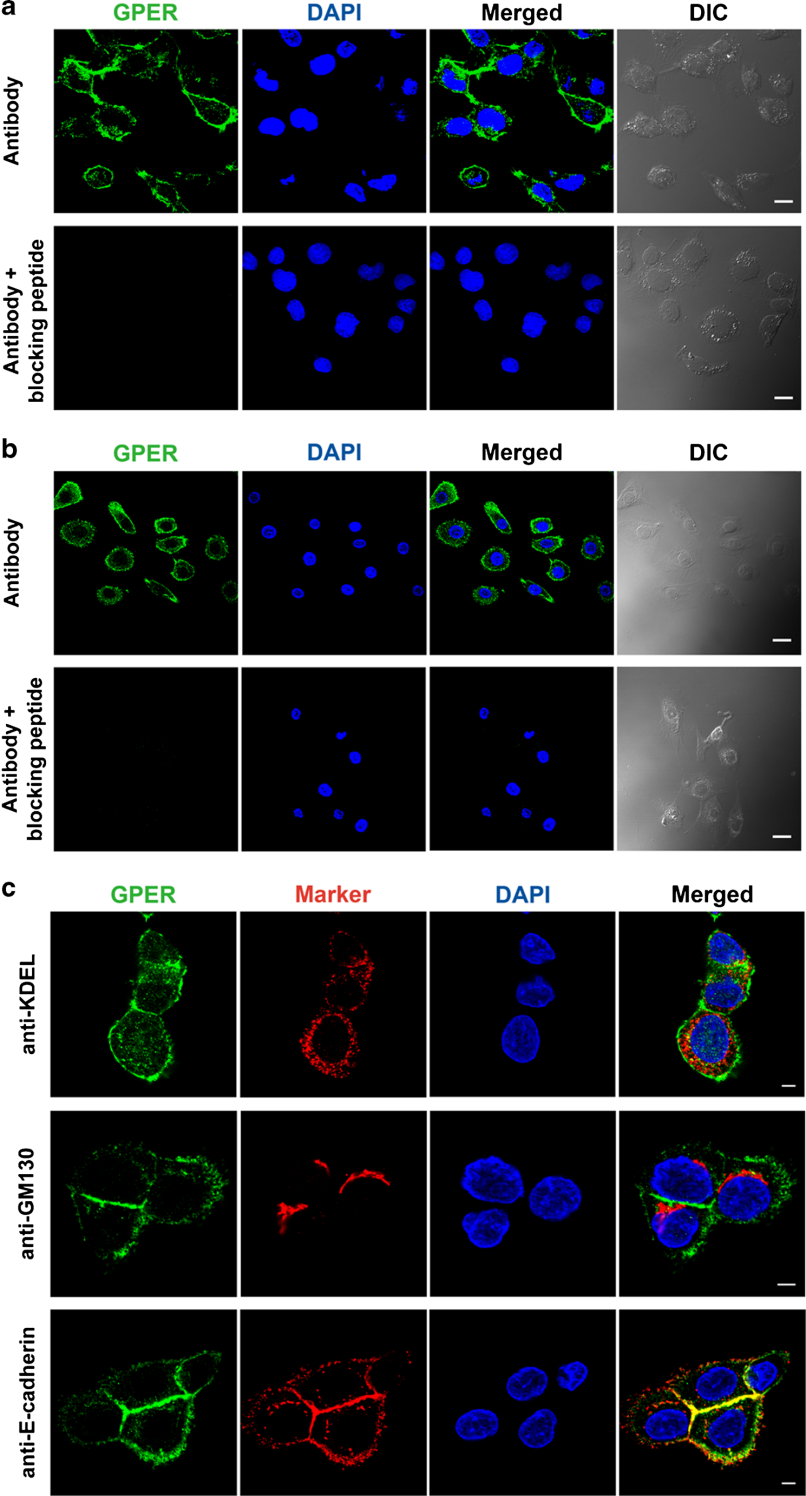
GPER localization in HBE cells. **a** 16HBE14o- cells immunostained with GPER antibody (sc-48525 N-15; *green*) and DAPI (nuclei; *blue*) (*upper row*). Bright-field photomicrograph shows the same field and cells. 16HBE14o- cells immunostained with GPER antibody preabsorbed with blocking peptide (*lower row*). *Scale bar*, 10 μm. **b** Similar GPER immunostaining results using primary HBE cells. *Scale bar*, 20 μm. **c** 16HBE14o- cells immunostained with GPER antibody and the ER marker, KDEL (*upper row*), the Golgi apparatus marker, GM130 (middle row), or the plasma membrane marker, E-cadherin (*lower row*). *Blue*, DAPI, nuclei. *Scale bar*, 10 μm. Images are representative of three to five independent experiments. (Color figure online)

Additionally, GPER expression was detected by immunofluorescence in 16HBE14o- cells (Fig. 2a). GPER was labeled with an Alexa Fluor 488-conjugated anti-GPER antibody, and nuclei were visualized by DAPI. There was no overlap between the GPER and nuclear signals. Moreover, no immunoreactivity was observed in sections incubated with GPER antibody preabsorbed with the blocking peptide, indicating that the GPER staining was specific. Similar immunostaining patterns were observed in primary HBE cells (Fig. 2b).

To further characterize the subcellular localization of GPER, double immunofluorescence labeling was used to determine possible colocalization between GPER and subcellular fractions, such as the endoplasmic reticulum (ER), Golgi apparatus, and plasma membrane, in 16HBE14o- cells (Fig. 2c). The results showed that GPER did not colocalize with Golgi (anti-GM130), while there was a very small amount of overlap between the GPER and the ER (anti-KDEL). In contrast, partial colocalization was observed between GPER and the plasma membrane (anti-E-cadherin) in 16HBE14o- cells.

### Inhibitory effects of E_2_ or the GPER agonist, G1, on nucleotide-induced Ca^2+^ signaling in HBE cells

**Fig. 3:**
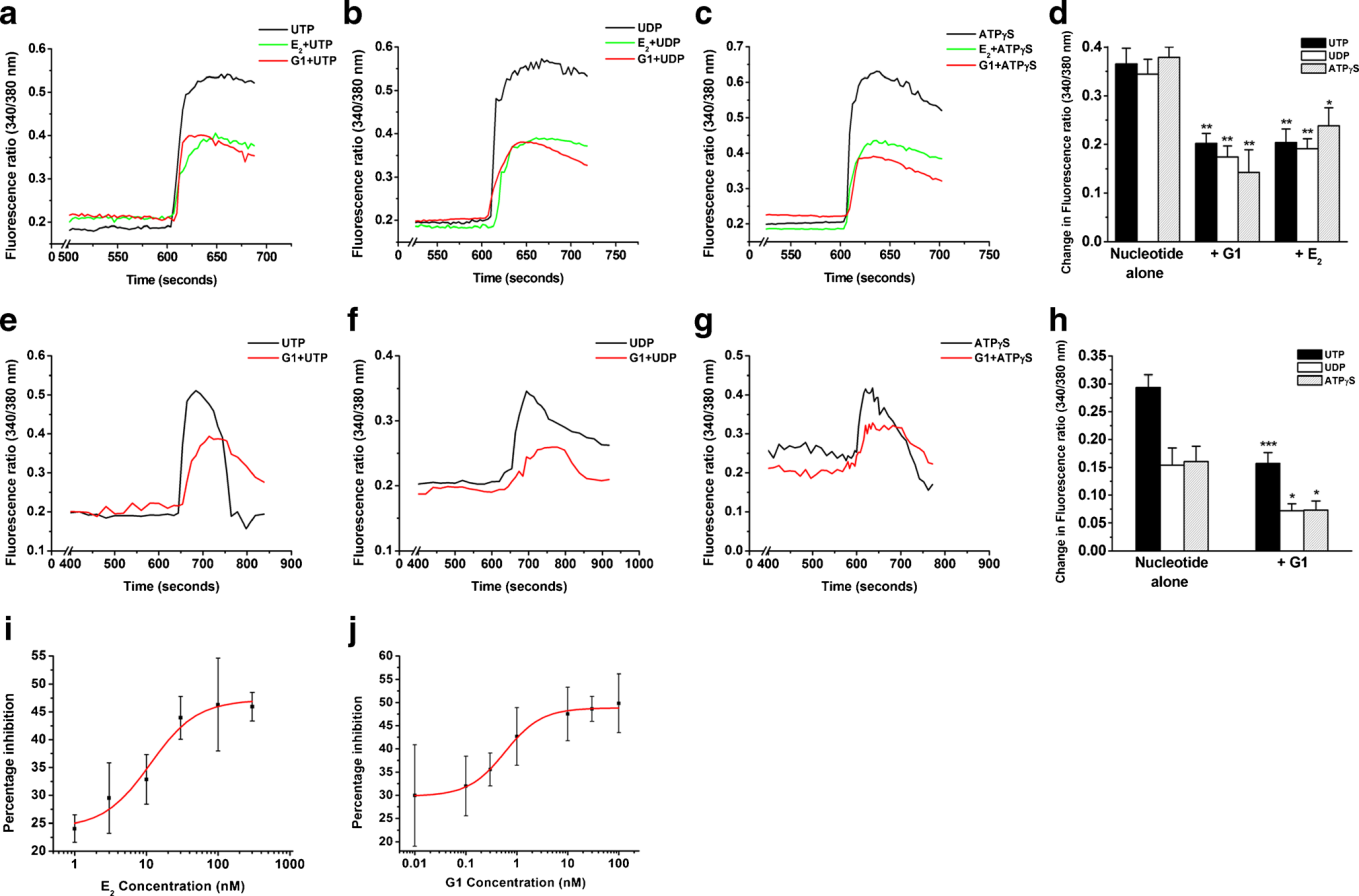
Inhibitory effects of E_2_ or G1 on nucleotide-induced increases in Ca^2+^ in HBE cells. 16HBE14o- (*n* = 4–6) were stimulated with 10-μM UTP (**a**), 100-μM UDP (**b**), or 10-μM ATPγS (**c**) in the absence (nucleotide alone) or presence of E_2_ (100 nM) or G1 (10 nM) for 10 min, and the maximal increase in the Fura-2 fluorescence ratio was quantified. **d** Statistical results for **a** to **c**. Similarly, primary HBE cells (*n* = 4–7) were stimulated with 10-μM UTP (**e**), 100-μM UDP (**f**), or 10-μM ATPγS (**g**) in the absence (nucleotide alone) or presence of G1 (10 nM) for 10 min, and **h** shows the summarized data. **i**, **j** UTP-induced Ca^2+^ increases in 16HBE14o- cells were inhibited in a concentration-dependent manner by E_2_ (**i**) or G1 (**h**) (*n* = 3–8). Data are expressed as the mean ± SEM. *P < 0.05, **P < 0.01, and ***P < 0.001 compared to nucleotide alone

Our previous study showed that P2Y receptors were expressed in airway epithelia and could be stimulated by nucleotides, such as ATP, UTP, and UDP, resulting in an increase in [Ca^2+^]_i_ [47]. Because it has been suggested that E_2_ inhibits the P2Y receptor-dependent Ca^2+^ signaling pathway [11], we examined whether activation of GPER had a similar inhibitory effect on P2Y receptor-mediated increases in Ca^2+^. In this study, both 16HBE14o- (Fig. 3a–d) and primary HBE (Fig. 3e–h) cells were treated with 100-nM E_2_ or 10-nM G1 for 10 min before being activated by 10-μM UTP (P2Y_2_ and P2Y_4_ agonist), 100-μM UDP (P2Y_6_ agonist), or 10-μM ATPγS (P2Y_2_ and P2Y_11_ agonist) [1]. The nucleotide-evoked increases in Ca^2+^ were inhibited by E_2_ and G1 with the percentages of inhibition, varying from 37.0 to 62.4 %. Both E_2_ and G1 were dissolved in DMSO, with a stock concentration 1000-fold higher than the final concentration. DMSO alone was used as vehicle control in all relevant experiments and did not produce any significant effect. Similar findings were obtained in primary HBE cells. The degree of G1-mediated inhibition of UTP-, UDP-, and ATPγS-induced increases in Ca^2+^ were 46.4, 53.4, and 54.6 %, respectively.

Various concentrations of E_2_ and G1 were used to examine their inhibitory effects on P2Y receptor-mediated Ca^2+^ signaling in 16HBE14o- cells. The 16HBE14o- cells were pretreated with E_2_ (1–300 nM) or G1 (0.01–100 nM) for 10 min and then stimulated by 10-μM UTP in the presence of E_2_ or G1. Both E_2_ and G1 inhibited the UTP-induced increases in Ca^2+^ in a concentration-dependent manner (Fig. 3i, j). The half maximal inhibitory concentration (IC_50_) values of E_2_ and G1 were 12.42 and 0.58 nM, respectively.

**Fig. 4:**
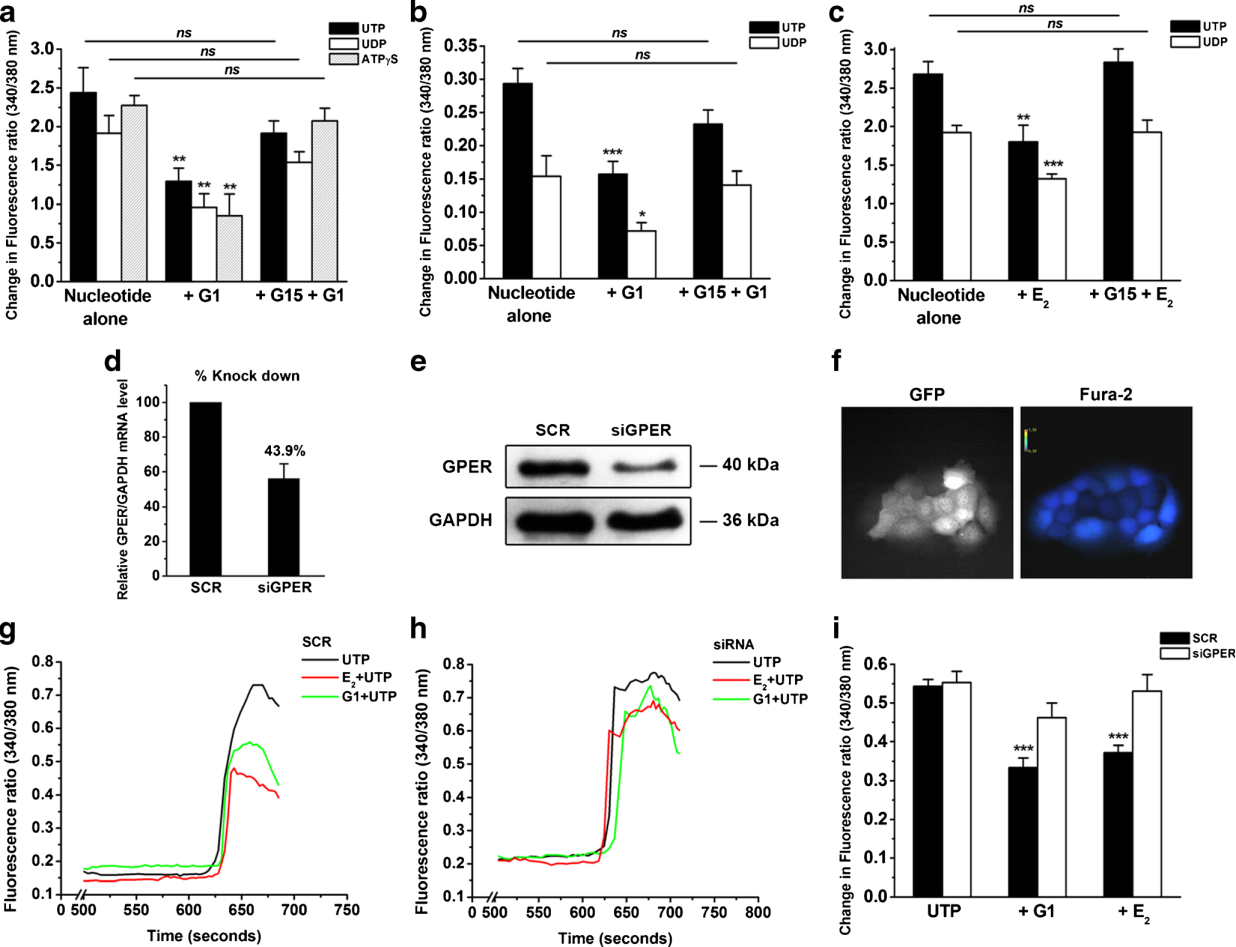
Reversal of E_2_- and G1-inhibited increases in Ca^2+^ via the GPER antagonist, G15, or GPER siRNA knockdown in HBE cells. **a** G15 (1 μM) reversed the inhibitory action of G1 (10 nM) on UTP (10 μM)-, UDP (100 μM)-, and ATPγS (10 μM)-induced increases in Ca^2+^ in 16HBE14o- cells (*n* = 4–5). **b** Similar results were obtained in primary HBE cells (*n* = 3–7). **c** G15 (1 μM) reversed the inhibitory effect of E_2_ (100 nM) on UTP (10 μM)- and UDP (100 μM)-induced increases in Ca^2+^ in 16HBE14o- cells (*n* = 4–6). **d** real-time PCR analyses of GPER expression in 16HBE14o- cells transfected with siRNA targeting GPER (siGPER) or scramble siRNA (SCR) (*n* = 5). **e** Protein band of GPER in 16HBE14o- cells transfected with siGPER or SCR. GAPDH was used as internal control (*n* = 4). **f** GFP and Fura-2 fluorescence in 16HBE14o- cells transfected with GFP-tagged siRNA. Fura-2 fluorescence intensity indicates Ca^2+^ density. **g**–**i** 16HBE14o- cells transfected with SCR or siGPER were stimulated with 10-μM UTP in the presence or absence of G1 (10 nM) for 10 min, and the maximal increase in the Fura-2 fluorescence ratio was quantified (*n* = 14–23 experiments in individual cells). Data are expressed as the mean ± SEM. *P < 0.05, **P < 0.01, and ***P < 0.001 compared to nucleotide alone

To confirm the specificity of the GPER-mediated inhibitory effect of G1, a newly developed GPER antagonist, G15 [14], was used. The 16HBE14o- cells were treated with 10-nM G1 in the presence or absence of 1-μM G15 for 10 min. The cells were then stimulated with 10-μM UTP. In the presence of G15, the inhibitory effect of G1 on the nucleotide-induced increase in Ca^2+^ was reversed in both 16HBE14o- (Fig. 4a) and primary HBE (Fig. 4b) cells. Furthermore, the inhibitory effect of E_2_ (100 nM) on the increase in Ca^2+^ was also reversed in the presence of G15 (Fig. 4c), indicating that E_2_ could inhibit nucleotide-induced Ca^2+^ signaling via GPER.

In addition to using a specific antagonist, we also used lentiviral vectors expressing siRNA to downregulate the *GPER* gene in 16HBE14o- cells. The knockdown efficiency of GPER was examined on both the mRNA and protein levels (Fig. 4d, e). The expression level of GPER in 16HBE14o- cells transfected with siRNA targeting GPER (siGPER) was 43.9 % lower than those transfected with a negative control siRNA encoding a scrambled sequence (SCR; Fig. 4f). The presence of GFP did not disturb Fura-2 fluorescence during the measurement of [Ca^2+^]_i_ [5]. The successfully transfected cells were selected for calcium measurements. The data show that 10-nM G1 inhibited UTP-induced increases in Ca^2+^ in the SCR control group (Fig. 4g), whereas no significant inhibitory effect was observed in the siGPER group treated with G1 (Fig. 4h, i). These results indicate that the inhibitory effect of G1 was mediated by GPER.

### Effects of E_2_ and G1 on nucleotide-induced Ca^2+^ release and influx

**Fig. 5:**
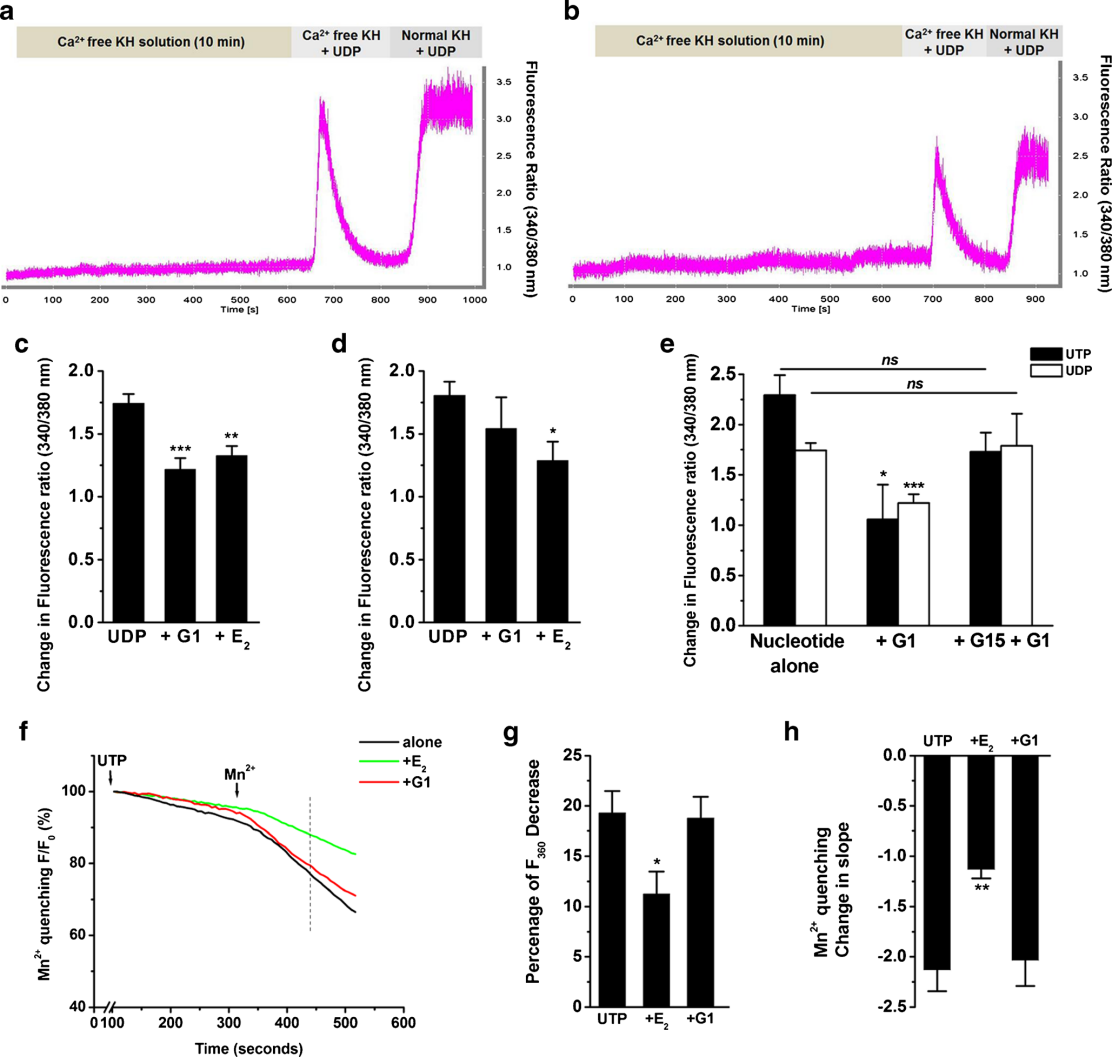
Inhibitory effects of E_2_ and G1 on the two phases of nucleotide-induced Ca^2+^ increase. **a**, **b** Representative recordings of Fura-2 fluorescence ratios in control (**a**) and E_2_-treated (**b**) cells. 16HBE14o- cells were superfused with nominally Ca^2+^-free solution for 10 min in the absence (control) or presence of E_2_ (100 nM) and then exposed to UDP (100 μM). Once [Ca^2+^]_i_ had returned to basal levels, external Ca^2+^ was subsequently restored (2.5 mM). **c**, **d** The inhibitory effect of E_2_ (**c**; 100 nM) and G1 (**d**; 10 nM) on UDP (100 μM)-induced Ca^2+^ release (first phase) and Ca^2+^ influx (second phase) in 16HBE14o- cells, respectively (*n* = 6–9). **e** Cells were stimulated with nucleotide (UTP or UDP) alone or in the presence of G1 (10 nM) with or without G15 (1 μM), and Ca^2+^ release (first phase) was quantified by Fura-2 fluorescence microscopy (*n* = 4–8). **f**–**h** Representative recordings and quantification of Mn^2+^ quenching of Fura-2 fluorescence. 16HBE14o- cells preincubated with E_2_ (100 nM) or G1 (10 nM) were stimulated with 10-μM UTP for 5 min before adding Mn^2+^ (1 mM). In **f**, maximal fluorescence (360 nm wavelength) was set to 100 % and fluorescence quench was measured 120 s after Mn^2+^ application. **g** Compiled data showing the percentage of fluorescence decrease 120 s after adding Mn^2+^ in cells treated with UTP alone or in the presence of E_2_ or G1. **h** Summarized data showing the change in slope before and after Mn^2+^ application. Data are expressed as the mean ± SEM. *P < 0.05, **P < 0.01, and ***P < 0.001 compared to nucleotide alone

Intracellular increases in calcium could result from the release of ER stores or influx through store-operated calcium (SOC) channels regulated by stromal interaction molecule 1 (STIM1) [26]. To examine the effects of E_2_ and G1 on P2Y receptor-activated intracellular Ca^2+^ release (first phase) and influx (second phase), epithelia were exposed to Ca^2+^-free solution in the presence or absence of G1 or E_2_ for 10 min and then stimulated with UDP or UTP together with G1 or E_2_. Subsequently, Ca^2+^ (2.5 mM) was added back to the perfusate to induce Ca^2+^ influx. Typical recordings of fluorescence ratios in control (Fig. 5a) and E_2_-treated (Fig. 5b) cells show that both 100-nM E_2_ and 10-nM G1 inhibited UDP (100 μM)-induced Ca^2+^ release in 16HBE14o- cells (Fig. 5c). However, only E_2_ inhibited UDP (100 μM)-induced Ca^2+^ influx (Fig. 5d). Moreover, the inhibitory effect of 10-nM G1 on UDP (100 μM)- or UTP (10 μM)-induced Ca^2+^ release (first phase) was reversed by 1-μM G15 (Fig. 5e). Taken together, these data demonstrate that E_2_ inhibited nucleotide-induced Ca^2+^ release and Ca^2+^ influx, whereas the GPER agonist, G1, only inhibited P2Y receptor-mediated Ca^2+^ release.

The manganese quench technique was also applied to report calcium influx through plasma membrane channels. The quenching of Fura-2 fluorescence by Mn^2+^ was measured at the Ca^2+^-independent excitation wavelength of Fura-2 (360 nm). When preincubating the cells with 100-nM E_2_ for 10 min, the rate of change on Mn^2+^ quenching was significantly decreased compared to untreated or G1 pretreated 16HBE14o- cells (Fig. 5f, g). Similarly, the percentage decrease of Fura-2 fluorescence 120 s after Mn^2+^ application also dropped significantly in E_2_ pretreated cells but not in G1 pretreated cells (Fig. 5h). These results indicate that E_2_, but not G1, blocked calcium influx through the plasma membrane.

Calcium release from the ER mainly occurs via inositol 1,4,5-trisphosphate receptors (IP_3_R) in human airway epithelial cells, including 16HBE14o- cells [40]. The activation of various subtypes of P2Y receptors causes an increase in Ca^2+^ via the phospholipase C (PLC)-IP_3_ signaling cascade [25]. Because the lifetime of IP_3_ within the cell before it is transformed into IP_2_ and IP_1_ is very short, IP_1_ accumulation levels can be used to represent IP_3_ levels in cells. To induce IP_1_ accumulation by activating the P2Y receptor-mediated signaling pathway in 16HBE14o- cells, 10-μM UTP (Fig. S1a) or 100-μM UDP (Fig. S1b) was used. Dimethyl sulfoxide (DMSO), the solvent used for E_2_ and G1, did not affect IP_1_ accumulation. UTP- or UDP-induced IP_1_ could be blocked by U73122 (10 μM), an inhibitor of PLC, whereas E_2_ (10 and 100 nM) or G1 (10 nM) had no effect on UTP- or UDP-induced IP_1_ accumulation.

**Fig. 6:**
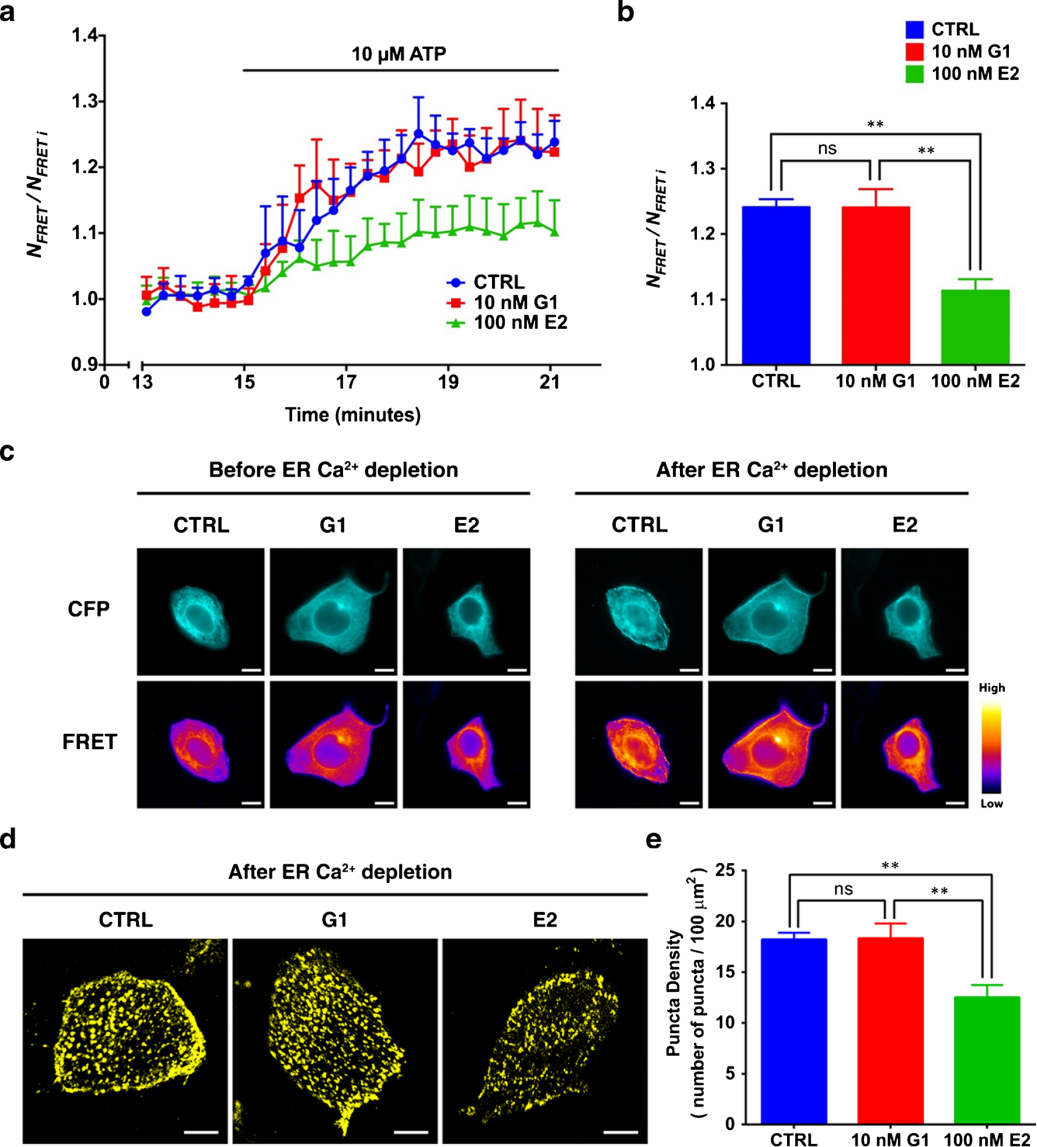
E_2_ impairs STIM1 oligomerization during Ca^2+^ depletion from the ER. **a** Time course of changes in *N*_*FRET*_ in 16HBE14o- cells co-transfected with CFP- and YFP-STIM1. Cells were pretreated with 10-nM G1 or 100-nM E_2_ in Ca^2+^-free HEPES-HBSS for 15 min. Cells pretreated with DMSO vehicle were used as controls (CTRL). Ca^2+^ from the ER was depleted by the addition of 10-μM ATP at *t* = 15 min. The experiment was repeated five times, and *N*_*FRET*_ of the cells was normalized to its initial value. **b** Statistical analysis of the changes in *N*_*FRET*_ at *t* = 20 min using one-way ANOVA with Tukey’s multiple comparison test, **P < 0.01. **c** Representative micrographs highlighting STIM1 localization before (*t* = 15 min) and after (*t* = 0 min) Ca^2+^ depletion from the ER. Images were captured in the CFP (*upper panel*) and FRET (*lower panel*) channels. *Scale bar*, 10 μm. **d** Representative confocal images depict the formation of YFP-STIM1 puncta in 16HBE14o- cells after ER Ca^2+^ was depleted by ATP. Cell were pretreated with DMSO (CTRL), 10-nM G1, or 100-nM E_2_ in Ca^2+^-free HEPES-HBSS for 15 min followed by ER Ca^2+^ depletion induced with 10-μM ATP. **e** STIM1 puncta were determined by ImageJ, and the amount of STIM1 puncta was summarized as mean ± SEM from six isolated cells obtained from three individual experiments of each treatment. Images were taken in ×60 oil immersion objective, and the *scale bar* represents 10 μm

The effects of E_2_ and G1 on SOC influx was determined by fluorescence resonance energy transfer (FRET) using 16HBE14o- cells co-expressing CFP- and YFP-STIM1. STIM1 proteins are known to undergo oligomerization in response to Ca^2+^ depletion in the ER [21]. The addition of ATP triggered Ca^2+^ release from the ER in 16HBE14o- cells, leading to the oligomerization of CFP- and YFP-STIM1, thus generating FRET signals (Fig. 6a). In controls and in cells pretreated with 10-nM G1, the addition of ATP elicited approximately a 20 % increase in *N*_*FRET*_, whereas in cells pretreated with 100-nM E_2_, the ATP-induced *N*_*FRET*_ signal was significantly attenuated (Fig. 6a, b). Prominent increase in FRET signal was observed due to the oligomerization and translocation of STIM1 near the plasma membrane in control and G1 pretreated 16HBE14o- cells (Fig. 6c). However, the FRET signal was greatly reduced in cells pretreated with E_2_ compared to controls and G1 pretreated cells (Fig. 6c). To further investigate that the increase in FRET signal was due to oligomerization of STIM1, we measured STIM1 puncta formation in response to ATP stimulation in the absence of extracellular Ca^2+^. Addition of ATP to control 16HBE14o- cells transfected with YFP-STIM1 generated significant amount of STIM1 puncta dots (Fig. 6d, e). ATP induced a comparable amount of STIM1 puncta formation in cells pretreated with 10-nM G1 (Fig. 6d, e). However, the STIM1 puncta formation was significantly reduced in cells pretreated with 100-nM E_2_ (Fig. 6d, e). Taken together, these results suggest that E_2_ attenuates SOC by inhibiting STIM1 oligomerization.

### E_2_- and G1-induced cAMP production

**Fig. 7:**
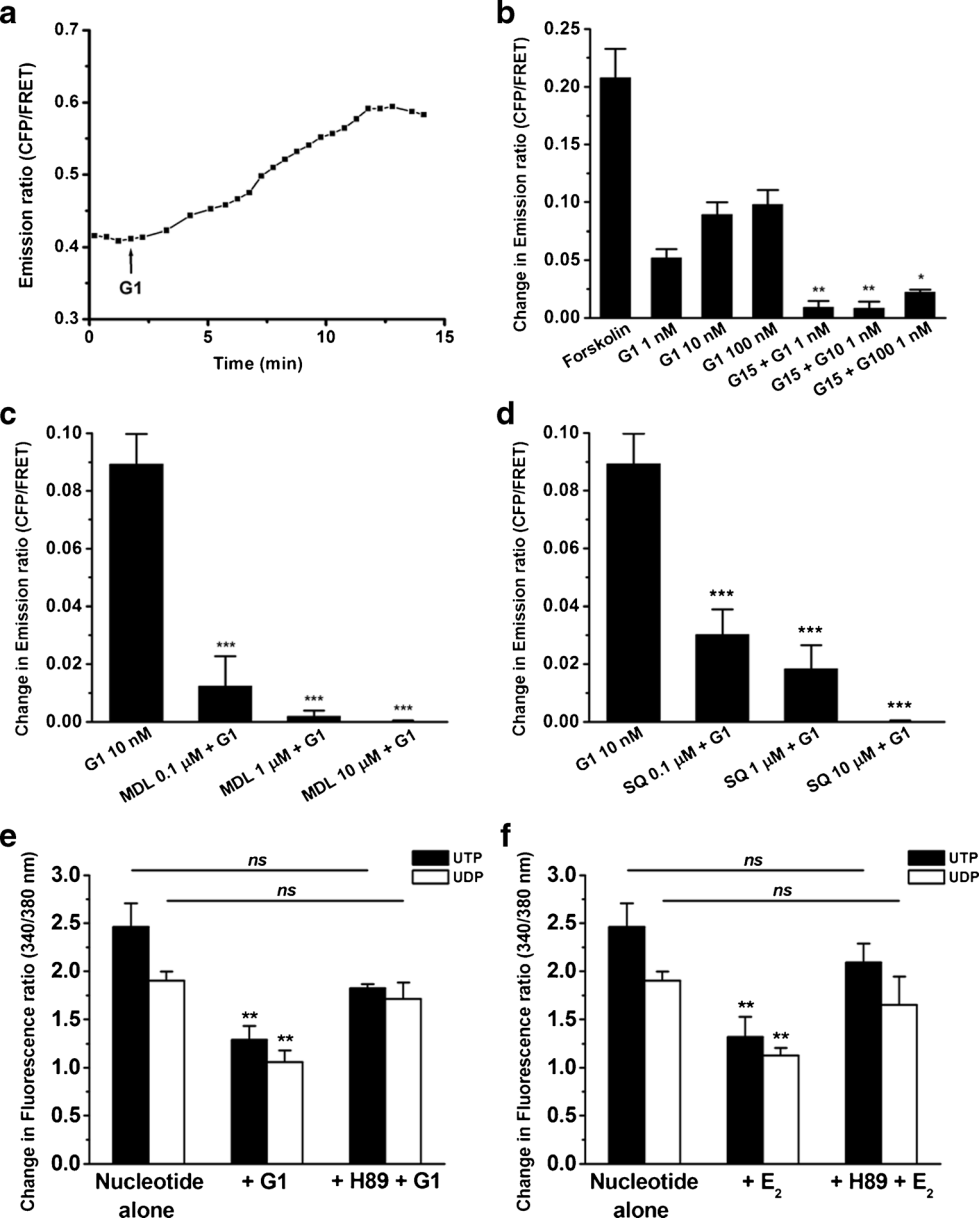
G1-stimulated cAMP mobilization and inhibition of increases in Ca^2+^ via cAMP-dependent PKA signaling in 16HBE14o- cells. **a** Real-time cAMP changes (represented by normalized CFP/FRET emission ratios) recorded in 16HBE14o- cells stimulated with G1 (10 nM). **b** 16HBE14o- cells were stimulated with different concentrations of G1 (1, 10, and 100 nM) alone or in the presence of G15 (1 μM). G15 was added 10 min before the addition of G1. Forskolin (25 μM) was used as a positive control to stimulate a maximal increase in cAMP levels (*n* = 4–8). **c**, **d** 16HBE14o- cells were treated with the adenylyl cyclase inhibitors, MDL 12330A or SQ 22536 (0.1, 1, and 10 μM), for 10 min followed by stimulation of the cells with G1 (10 nM) (*n* = 4–6). **e**, **f** Cells were treated with E_2_ (100 nM) or G1 (10 nM) in the presence or absence of the cAMP-dependent PKA inhibitor, H89 (10 μM), for 10 min, followed by stimulation of the cells with UTP (10 μM) or UDP (100 μM) (*n* = 3–5). Data are expressed as the mean ± SEM. *P < 0.05, **P < 0.01, and ***P < 0.001 compared to G1 or nucleotide alone

GPER activation may initiate the cAMP and protein kinase A (PKA) signaling pathways [50]. Thus, real-time cAMP levels were monitored in 16HBE14o- cells stimulated by G1. After the addition of 10-nM G1 to 16HBE14o- cells, the cAMP levels increased (Fig. 7a). This stimulatory effect of G1 on cAMP levels was blocked by the GPER antagonist, G15 (Fig. 7b), indicating that the effect was specific. Similarly, E_2_ (100 nM) also induced an increase in cAMP levels (change in emission ratio = 0.12 ± 0.02, *n* = 5). When the cells were treated with 1-μM G15 for 10 min before the addition of various concentrations of G1, cAMP production was significantly inhibited compared to G1 alone.

To demonstrate the involvement of adenylyl cyclase (AC) in cAMP production, two AC inhibitors, MDL 12330A and SQ 22536, were used. G1-induced cAMP production was significantly inhibited by 10-min treatments with MDL 12330A (0.1, 1, and 10 μM) in a concentration-dependent manner (Fig. 7c). Similar results were obtained with SQ 22536 (Fig. 7d). These results suggest that GPER is coupled to the activation of AC, likely via the Gs alpha subunit, to stimulate an increase in cAMP levels in 16HBE14o- cells.

### The role of PKA in E_2_- and G1-mediated inhibition of calcium increases

We next determined whether the cAMP-dependent pathway was involved in the inhibitory effects of E_2_ and G1 on the P2Y-induced increase in Ca^2+^ in 16HBE14o- cells. H89, a PKA inhibitor, was used to inhibit downstream signaling targets of cAMP. The inhibitory effects of G1 (10 nM) on 10-μM UTP- or 100-μM UDP-induced increases in Ca^2+^ were reversed by co-incubation of the cells with H89 (10 μM) for 10 min (Fig. 7e). Similar results were obtained with 100-nM E_2_ (Fig. 7f). These results demonstrate that the inhibitory effects of E_2_ and G1 on P2Y receptor-induced Ca^2+^ signaling are mediated via the activation of a cAMP-dependent PKA pathway.

### Effects of the GPER agonist, G1, on nucleotide- or poly-l-arginine-induced cytokine production in HBE cells

**Fig. 8:**
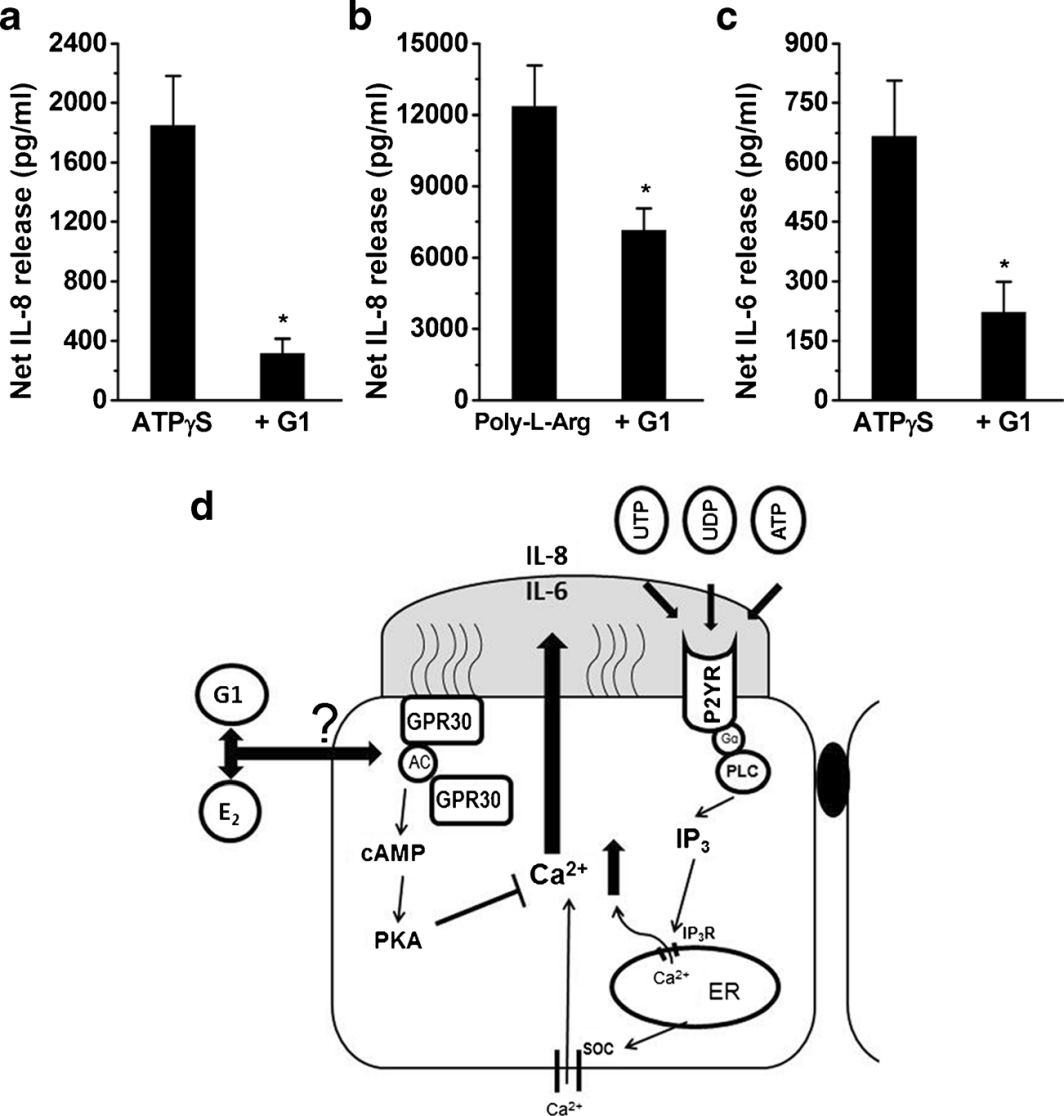
G1 inhibited nucleotide-stimulated IL-8 and IL-6 release in 16HBE14o- cells. **a**–**c** 16HBE14o- cells were treated with ATPγS (**a**, **c**; 10 μM) or poly-l-arginine (**b**; 1 μM) for 6 h in the presence or absence of G1 (10 nM). The supernatant was then collected, and IL-8 or IL-6 was quantified using an ELISA kit. The levels of IL-6 and IL-8 were corrected against vehicle control alone and expressed as the net release. Data are expressed as the mean ± SEM. *P < 0.05 compared to ATPγS or poly-l-arginine alone (*n* = 3–8). **d** Schematic diagram showing the GPER function in regulating P2Y receptor-mediated Ca^2+^ signaling and cytokine secretion in human bronchial epithelia. In HBE cells, nucleotides, such as UTP, UDP, and ATP, activate P2Y receptors to modulate intracellular increases in Ca^2+^, which lead to the release of proinflammatory cytokines, such as IL-8 and IL-6. Activation of GPER by E_2_ or its specific agonist, G1, attenuates both the nucleotide-evoked increases in Ca^2+^ and the cytokine secretion. This inhibitory effect is likely due to the activation of a GPER-mediated cAMP/PKA pathway. Note that whether G1 and E_2_ can translocate through the plasma membrane to act on the receptor that needs to be verified

Our previous study indicates that the levels of two proinflammatory cytokines, interleukin 8 (IL-8) and IL-6, increase significantly after airway bronchial epithelial cells are stimulated by extracellular nucleotides when the cells are damaged by poly-l-arginine [20]. Therefore, the effects of G1 on nucleotide- or poly-l-arginine-induced IL-8 and IL-6 production were determined in HBE cells. ATPγS (10 μM) or poly-l-arginine (3 μM) was incubated with the cells for 6 h in the presence or absence of G1 (10 nM). Significant inhibition of ATPγS- or poly-l-arginine-stimulated IL-8 release was observed under these conditions (Fig. 8a, b). The addition of 10-nM G1 also showed a significant inhibitory effect on ATPγS-stimulated IL-6 release in 16HBE14o- cells (Fig. 8c). These results indicate that GPER may play an important role in inhibiting proinflammatory cytokine secretion stimulated by P2Y receptor activation in HBE cells.

## Discussion

GPER is known to play important roles in multiple tissues, including the heart, brain, lung, liver, skeletal muscle, and kidney [33]. However, few reports have described the expression and function of GPER in airway epithelial cells. Only one study described the relatively high expression of GPER in human nonsmall cell, lung cancer cell lines compared to immortalized normal lung bronchial epithelial cells [23]. This study therefore reports for the first time the expression and function of GPER as an anti-inflammatory component in human bronchial epithelia and highlights that GPER likely serves this role through its opposing effects on the proinflammatory pathway activated by the P2Y receptors in inflamed airway epithelia.

The mRNA and protein expression levels of GPER in primary HBE and 16HBE14o- cells were confirmed by RT-PCR and Western blotting, respectively. Although GPER localizes to the ER [35] and plasma membrane [17], its subcellular localization is still controversial. Overall, the localization of GPER appears to vary depending on the cell type. Our immunofluorescence studies showed partial colocalization between the GPER and the plasma membrane with no overlap between GPER and Golgi, or nucleus. Besides, a very small amount of GPER was localized in ER. These results were similar to those observed in osteocyte-like MLO-Y4 cells and transfected HEK-293 cells [17, 34]. Different tissues or cell types may have different subcellular GPER localizations, which may be due to the different roles GPER plays in various cell types, such as cell proliferation, apoptosis, and immune responses [27]. However, it should be noted that, even in the same cells, GPER could change its location via endocytotic processes. Two reports have demonstrated that GPER can be trafficked intracellularly from the plasma membrane [8, 38]. This unique mechanism could decrease the amount of GPER at the plasma membrane and protect cells from chronic signaling. Thus, we could not exclude this possibility, but the exact details of this intriguing membrane receptor trafficking pathway in human bronchial epithelia require further study.

In this study, activation of GPER by G1 did not alter basal [Ca^2+^]_i_ levels, but it did significantly inhibit P2Y receptor-mediated increases in Ca^2+^. This inhibitory effect was not P2Y receptor subtype-specific, because G1 inhibited the Ca^2+^ responses elicited by different P2Y receptor subtype ligands (e.g., UTP, UDP, and ATPγS). The inhibitory effect could be reversed when pretreating with GPER-specific antagonist, G15. Besides, siRNA knockdown of GPER was also applied to further confirm the role of GPER. Various transfect reagents (e.g., lipofectamine 2000, lipofectamine RNAiMax, siPORT NeoFX, DharmaFECT 1 and 4) have been applied in our experiment for GPER silencing; however, the knockdown efficiency was low and inconsistent (data not shown). Therefore, lentiviral-mediated silencing approach was adopted and a stable silenced cell line was generated with better knockdown efficiency on GPER. To reveal whether G1 inhibited intracellular increases in Ca^2+^ by blocking Ca^2+^ release and/or Ca^2+^ influx, we characterized the two phases of Ca^2+^ increase by perfusing cells with a Ca^2+^-free Krebs-Henseleit Buffer, followed by a Ca^2+^-containing solution. The results showed that E_2_ inhibited both P2Y receptor-mediated Ca^2+^ release and Ca^2+^ influx, whereas G1 only inhibited Ca^2+^ release. However, the observed difference in Ca^2+^ signals could be due to regulation of Ca^2+^ pumps. The inhibitory effect of E_2_ on Ca^2+^ influx was further confirmed by the data obtained from Mn^2+^ quench experiments. Taken together, these data suggest that the activation of GPER only inhibits P2Y-activated IP_3_-mediated Ca^2+^ release, whereas classical E_2_ receptors activated by E_2_ played a role in regulating Ca^2+^ influx. FRET microscopy further confirmed that the inhibitory effect of E_2_, but not G1, on SOC influx was due to the inhibition of STIM1 oligomerization. Our findings were similar to those reported recently [39], which showed that E_2_ can signal nongenomically by inhibiting basal phosphorylation of STIM1, leading to a reduction of SOC entry in human airway cells.

The inhibition of Ca^2+^ release by G1 could occur via different pathways. For example, GPER activation might block the activity of PLCβ to diminish the synthesis of IP_3_, inhibit the activity of IP_3_R to release Ca^2+^ from ER, or stimulate Ca^2+^ uptake into stores by activating the endoplasmic Ca^2+^-ATPase pump. To investigate the detailed mechanism underlying the inhibitory effects of E_2_ and G1 on P2Y receptor-mediated Ca^2+^ mobilization, we conducted another series of studies to examine whether E_2_ or G1 could inhibit IP_3_ production. We measured IP_1_ accumulation to determine IP_3_ levels. IP_1_ accumulation induced by both UDP and UTP was significantly inhibited by the PLC inhibitor, U73122, whereas the addition of E_2_ or G1 showed no inhibitory effects. This result indicates that the E_2_- or G1-mediated inhibition of P2Y receptor-mediated Ca^2+^ signaling was not related to a change in IP_3_ levels. Thus, the observed inhibition may be due to the inhibition of IP_3_ independent of Ca^2+^ release. Alternatively, it may be due to an unidentified signaling pathway or molecule that interferes with the interaction between IP_3_ and IP_3_R [2].

GPER couples to different signaling pathway(s), including the cAMP/PKA pathway [50]. Our data suggest that GPER in human bronchial epithelia was coupled to AC, resulting in an increase in cAMP levels. Notably, blocking the downstream target of cAMP with H89 reversed the inhibitory effect of G1 on P2Y receptor-mediated Ca^2+^ signaling, and cAMP-dependent protein kinase reportedly inhibits IP_3_-induced Ca^2+^ release in human bone marrow cells [42]. The cAMP/PKA pathway may inhibit receptor-operated calcium entry (ROCE) via transient receptor potential canonical channel 6 (TRPC6). TRPC6 is expressed in both undifferentiated and differentiated primary HBE cells [12]. Calcium influx mediated by TRPC6 is functionally coupled to calcium-activated chloride channel activity in human airway epithelial cells [4] and can be regulated by P2Y receptor activation in mouse podocytes [37]. A recent study suggests that the cAMP/PKA signaling pathway can inhibit endothelin type A receptor-mediated ROCE via TRPC6 by phosphorylation of Ser28 site in human embryonic kidney 293 cells [22]. Although G1 did not have any significant effect on nucleotide-mediated Ca^2+^ influx, we did not explicitly examine P2Y receptor-mediated ROCE in this study. It would be interesting for future research to examine if GPR30 can inhibit P2Y receptor-mediated ROCE via TRPC6 in human airway epithelia. In addition to PKA, Epac is another downstream target of cAMP that transduces diverse cellular actions [7, 9]. The cAMP increases evoked by G1 are sufficient to activate Epac. Interestingly, our previous study demonstrates that both Epac 1 and Epac 2 are expressed in 16HBE14o- cells [24]. Therefore, we could not exclude the possibility that some of the observed inhibitory effects were mediated through activation of Epac. Moreover, our recent study demonstrates that the proinflammatory effect of nucleotides is mediated via an increase in [Ca^2+^]_i_ after P2Y receptor activation. Treating 16HBE14o- cells with the intracellular Ca^2+^ chelator, BAPTA-AM, but not H89, inhibited P2Y receptor-mediated IL-6 and IL-8 secretion [20]. Taken together, GPER likely inhibits the P2Y receptor-mediated inflammatory response by downregulating [Ca^2+^]_i_ in human airway epithelia. A recent study reported that Ca^2+^-dependent calmodulin can regulate GPER-dependent signaling at the receptor level [44]. Therefore, a P2Y receptor-mediated increase in Ca^2+^ could, in turn, regulate GPER function, but the details of the possible cross talk between the two receptors require further investigation. 16HBE14o- cells were cultured in MEM without phenol red in some experiments since phenol red may serve as a weak estrogen mimic. However, no significant differences have been observed in terms of GPER expression and the inhibitory effect of G1 on P2Y receptor-mediated cytokine secretion and Ca^2+^ increase (data not shown) in cells cultured in MEM with or without phenol red.

In summary, this study characterizes the expression, localization, and role of GPER, as well as its interaction with P2Y receptors, that were co-expressed in human bronchial epithelia. Activation of GPER by E_2_ or its specific agonist, G1, rapidly attenuated a nucleotide-evoked increase in Ca^2+^, whereas the specific GPER antagonist, G15, reversed this GPER-mediated inhibition. Furthermore, E_2_ and G1 also inhibited nucleotide-induced cytokine release. The inhibitory effects on P2Y receptor-mediated Ca^2+^ mobilization and cytokine secretion are likely due to GPER-mediated activation of a cAMP-dependent PKA pathway in human bronchial epithelia (Fig. 8d).

## Electronic supplementary material

Below is the link to the electronic supplementary material.

ESM 1 (DOCX 1437 kb)
