# Supplementary material for: G protein-coupled estrogen receptor inhibits the P2Y receptor-mediated Ca2+ signaling pathway in human airway epithelia
Source: Pflugers Arch. 2016 Jun 6;468:1489–503. doi: 10.1007/s00424-016-1840-7 (PMC4951515; doi:10.1007/s00424-016-1840-7)
Supplement: Supplementary file 1 — (DOCX 1437 kb) [file 424_2016_1840_MOESM1_ESM.docx]

**Supplementary Materials for**

**GPER inhibits the P2Y receptor-mediated Ca^2+^ signaling pathway in human airway epithelia**

Yuan Hao, Alison W. Chow, Wallace C. Yip, Chi H. Li, Tai F. Wan, Benjamin C. Tong, King H. Cheung, Wood Y. Chan, Yangchao Chen, Christopher H. Cheng, and Wing H. Ko*

*Corresponding author. E-mail: whko@cuhk.edu.hk; Phone: +852 39436781

**Supplementary Figure 1**


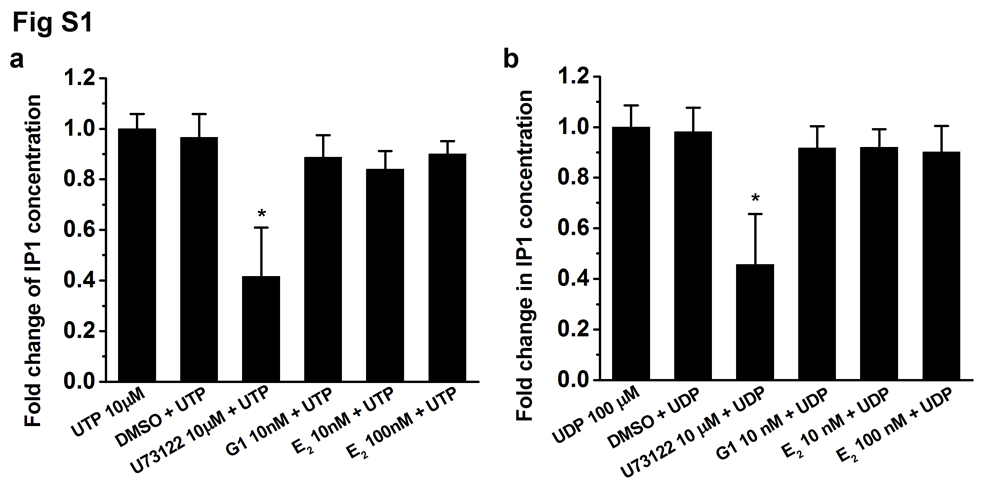


**Fig. S1. E_2_ and G1 did not inhibit nucleotide-induced inositol-1-phoshate (IP_1_) accumulation.** In 16HBE14o- cells, IP_1_ production stimulated by UTP (10 µM) or UDP (100 µM) for 1 h was blocked by the PLC inhibitor, U73122 (10 µM), but not by E_2_ (10 nM and 100 nM) or G1 (10 nM), in 16HBE14o- cells (n = 4 - 6). Data are expressed as the mean ± SEM. * *P* < 0.05 compared to nucleotide alone.
